# Longitudinal Changes in Sleep: Associations with Shifts in Circulating Cytokines and Emotional Distress in a Cancer Survivor Population

**DOI:** 10.1007/s12529-020-09950-0

**Published:** 2021-02-17

**Authors:** Jo A. Tucker, Kathryn Osann, Susie Hsieh, Aditi Wahi, Bradley J. Monk, Lari Wenzel, Edward L. Nelson

**Affiliations:** 1 Department of Medicine, Division of Hematology/Oncology, University of California, Irvine, CA USA; 2 Chao Family Comprehensive Cancer Center, University of California, Irvine, CA USA; 3 Department of Medicine, Division of General Internal Medicine, University of California, Irvine, CA USA; 4 Arizona Oncology, US Oncology Network, University of Arizona College of Medicine, Creighton University School of Medicine, Phoenix, AZ USA; 5 Program in Public Health, University of California, Irvine, CA USA; 6 Institute for Immunology, University of California, Irvine, CA 92617 USA

**Keywords:** Sleep, Cytokine, Actigraphy, Cancer survivors, PNI, Emotional distress

## Abstract

**Background:**

Sleep disturbances are associated with numerous mood disorders. Similarly, anxiety and depression are associated with modulation of the psychoneuroimmune (PNI) axis. This study hypothesized that changes in both monitored and self-reported measures of sleep would relate to changes in circulating cytokine levels in an emotionally distressed population of cervical cancer survivors.

**Methods:**

Biospecimens, patient-reported outcome (PRO) measures, and actigraphy were collected from cervical cancer survivors enrolled in a biobehavioral clinical trial. Longitudinal changes over a 4-month period were examined. Sleep time measured by actigraphy and PRO were analyzed for correlative changes with emotional distress and serum cytokines (n = 71).

**Results:**

Longitudinal change in the actigraph measure of sleep time was inversely associated with changes in depression and anxiety (test for linear trend, *p* = 0.02 and *p* = 0.05 respectively), as well as acute-phase response/pro-inflammatory cytokines (test for linear trend, *p* = 0.003, interleukin (IL)-2; 0.022, IL-1β; 0.0002, IL-6; and 0.049, tumor necrosis factor α). Conversely, changes in self-reported sleep problems were related to an increase in depression and anxiety (*p* = 0.001 and *p* = 0.01 respectively), the T helper 2 (Th2) cytokine IL-5 (*p* = 0.027), and the counter-regulatory cytokine IL-10 (0.016).

**Conclusion:**

This study showed that an increase in sleep time or decrease in sleep problems corresponded with a reduction in self-reported emotional distress and attenuation of pro-inflammatory, Th2, and counter-regulatory cytokines. Our results support sleep measurement as a meaningful biobehavioral variable in cancer survivorship. This study also indicates that sleep investigators should be aware that choice of methodology might influence concordance with different classes of immune parameters.

## Introduction

The relationship between sleep and emotional distress is complex [1–8]. Sleep disturbances have been described as being predictive of subsequent development of depression or other psychological diagnoses [2, 3]. Sleep problems may also be a consequence and downstream effect of the psychological stress response [9–12], and in some situations, a mediator of the pyschoneuroimmune (PNI) axis derangements associated with the chronic stress response [7–9, 13]. Evidence points to immune activation and resulting inflammation as a potential physiological link between distress and sleep.

Cervical cancer survivors report a lower quality of life (QOL) and significantly higher than average levels of depression and anxiety [14]. We have previously identified an association between improved QOL and a shift in the T helper (Th) cytokine profile toward a suppressive Th2 type in cervical cancer survivors [15, 16]. The presence of pro-inflammatory cytokines, such as IL-1 and TNFα, as well as suppressive cytokines like IL-4 and IL-10, has been shown to be inhibitory to normal sleep [17, 18]. Several studies in cancer patients and survivors have shown that cytokine milieus change in relation to sleep, particularly suppressive cytokines like IL-4 and IL-10 [19–22]. Considering the link between sleep, inflammation, and emotional distress, we hypothesized that within the cervical cancer survivor population, longitudinal changes in sleep parameters would be associated with both modulation of circulating cytokine levels and patient-reported outcome (PRO) measures of psychological distress as a surrogate for chronic psychological stress.

In sleep studies, many consider polysomnography (PSG) to be the “gold standard,” but it can be too expensive and labor-intensive for some studies. Alternatives include other objective measures, such as actigraphy, as well as subjective measures, such as sleep logs or PROs. Actigraphy provides a precise measurement of the motion of the part of the body to which a device is attached. It was found to be a valid method of detecting sleep time in normal adult populations when compared with PSG but less reliable when sleep became more disturbed [23, 24] or a motion disorder existed. Actigraphy does not provide appropriate information for diagnosis of sleep disorders, but it can be useful for screening and treatment assessment [25]. While several studies report that self-reported sleep overestimates some sleep parameters when compared with actigraphy [26–28], other studies have shown correlation [29, 30].

Sleep assessment in the survivors of several cancer types has been well documented, but to date, no studies have been published on sleep specifically in cervical cancer survivors. One study of sleep status in cervical cancer patients undergoing adjuvant therapy showed that treatment affected sleep quality, but psychological distress, depression, and anxiety were also factors associated with poor sleep quality [31]. The dearth of information about sleep assessment in this population prompted this study on examining how a subjective measure, a PRO, and an objective measure, actigraphy, correlate in this population and how they each relate to other PNI parameters, such as emotional distress and circulating immune cytokines.

## Methods

### Subjects

PRO data and biological samples were obtained from participants enrolled in a randomized biobehavioral clinical trial of cervical cancer survivors. Study design, recruitment, and baseline characteristics for this group have been previously published [15]. Participants were eligible if they had stage I to IVA disease and had completed definitive treatment at least 2 months prior to enrollment. Patients were excluded for treatment with biologic response modifiers or prior immunotherapy within 4 weeks of study enrollment, use of investigational drugs within 30 days, corticosteroids, or immunosuppression. The institutional review boards of both the University of California Irvine and California Cancer Registries approved the protocol. All participants provided written informed consent. All procedures performed in studies involving human participants were in accordance with the ethical standards of the institutional and/or national research committee and with the 1964 Helsinki declaration and its later amendments or comparable ethical standards.

Participants were randomized to usual care or a psychosocial telephone counseling intervention, which did not specifically target improvement in sleep patterns. Data and specimens for this study were collected at enrollment (T1) and 4 months post-enrollment (T2), after completion of the intervention (spanning 10 weeks). Participant characteristics are displayed in Table 1.

**Table 1:** Participant characteristics

Characteristics	No. of participants
Race/ethnicity	
White/non-Hispanic	59
Hispanic	11
Other	1
Stage	
I	53
II-IVA	18
Treatment	
Surgery only	34.79
Radiation only	5.43
Chemo ± radiation	30.7
Age at diagnosis	years
mean	45.2

### Study Measures

Participants completed the Patient-Reported Outcomes Measurement Information System (PROMIS) emotional distress, depression, and anxiety short forms [32]. The scale score was computed using proration when more than 50% of items were answered in consensus with PROMIS scoring convention (http://www.nihpromis.org). A high score connotes more depression or anxiety. This measure is considered a proxy for chronic psychological stress in this setting. The Medical Outcomes Study (MOS)-Sleep is a 12-item self-report instrument with multiple subscales [33]. These include (i) sleep disturbance; (ii) sleep adequacy; (iii) daytime somnolence; (iv) snoring; (v) awaken short of breath or with headache; (vi) quantity of sleep; and (vii) sleep problems. The sleep problems index (SLP9) is a summary score derived from the average of 9 items, transformed to a 0–100 range. Items are administered using a past 4-week recall interval. Higher scores on subscales with the exception of sleep adequacy indicate more sleep problems. Internal consistency for the sleep problems index is reported at 0.85 [33].

### Actigraphy

The Actiwatch 16 (Respironics, Bend, OR) wrist monitor was used to record objective sleep data. Participants were instructed to wear an Actiwatch the night before blood and PROs were collected at each time point, and to record sleep and wake time. The Actiwatch was collected and transported by the phlebotomist the day following the nighttime data collection. Data was extracted and analyzed using Actiware 5 software (Respironics, Bend, OR). The sleep interval was set automatically with the parameter of 10-min immobility for sleep onset and sleep end and recorded in 30-s epochs. Subjects for whom a sleep interval could not be determined at T1 or T2, due to Actiwatch misuse, were excluded (*n* = 36). In the remaining subjects, sleep onset and sleep end could be reconciled within 2 h of the self-reported data. The analysis of the actigraph data yielded various sleep parameters, defined as follows: sleep duration = minutes from sleep onset to sleep end; total AC = sum of all activity counts in the sleep duration; sleep time = total minutes scored as sleep; no. of sleep bouts = number of continuous blocks of sleep; avg sleep bout = average duration of the sleep bouts; and sleep efficiency = sleep time/sleep duration.

### Plasma Cytokines

Circulating cytokines were determined as previously described [15]. Briefly, plasma was collected from whole blood with EDTA anticoagulant by centrifugation, divided into aliquots and stored at − 80 °C until batched analysis. Circulating levels of cytokines were determined with the Milliplex® MAP Human High Sensitivity Cytokine/Chemokine Magnetic Bead Kit (EMD Millipore, Billerica, MA), which includes cytokines IFNγ, IL-1β, IL-2, IL-4, IL-5, IL-6, IL-10, IL-12p70, IL-13, and TNFα. Samples were prepared in accordance with the manufacturer’s instruction and tested in duplicate, with an individual’s samples tested on the same plate. Samples with lipemia or evidence of hemolysis were excluded. Data was collected on a MAGPIX® (Luminex, Austin, TX) and analyzed using Milliplex Analyst 5.1 software (EMD Millipore, Billerica, MA). Cytokine levels were determined in pg/ml from five-parameter log curves and logarithmically transformed.

### Statistics

Statistics were performed with Prism (GraphPad Software, La Jolla, CA) and confirmed by SAS (SAS Institute, Cary, NC). Correlations were reported as Spearman’s rho. Data was analyzed as a matrix, and Bonferroni’s correction was used for multiple comparisons. To investigate associations between change in sleep measures and change in cytokines, subjects were categorized into 3 subgroups: a decrease greater than or equal to one standard deviation (SD) from the mean, change less than one SD from the mean in either direction, or an increase greater than or equal to one SD from the mean, in either actigraphy-monitored sleep time or the SLP9. An F-test for linear trend was used to test for trends in cytokine change across subgroups defined by change in sleep. Significance was set at *p* < 0.05.

## Results

### Subjects and Measures

In order to examine the relationship of sleep with cytokines and PRO, we compared data from participants that took part in a randomized biobehavioral clinical trial of cervical cancer survivors. There were 204 participants in the trial, but due to issues such as problems with blood collection and actigraph misuse, not all data was available for all participants. Only 71 participants had complete actigraph, PRO, and cytokine data at both time points 1 and 2, and so were included in this study. A comparison of treatment groups, psychosocial telephone counseling (PTC) and usual care (UC), within these participants yielded no significant difference in any parameters in the longitudinal change between time points 1 and 2 (Supplemental Table 1), or at time points 1 or 2 (Supplemental Tables 2 and 3).

The clinical trial study did find differences in the longitudinal changes between treatment groups in FACT-Cx, FACT-Cx Additional Concerns, GPC, and PROMIS Depression. The fact that we do not see those differences in this subset of participants is likely due to the exclusion of over half of the participants due to missing data but could be because of another confounding factor or moderator. As the purpose of this study was to understand how sleep measures related to other data in this population rather than the intervention, further studies focused on comparison of sleep assessment methods without separating participants based on treatment group.

### The Relationship of Monitored and Self-Reported Sleep Measures in a Population of Cervical Cancer Survivors

Sleep was assessed using two methods, both actigraphy and a self-reported measure. The Actiwatch 16 actigraph device was worn on the wrist for one night immediately prior to PRO and biologics being collected at each time point. Actiwatch data was extracted and analyzed using Actiware 5 software. Epochs were set at 30 s and sleep onset at 10 min mobility. Self-reported sleep was determined by use of The Medical Outcomes Study (MOS)-Sleep. In addition to the fundamental difference in these two measurement modalities (PRO and actigraphy), it is important to note that actigraphic data was a single “snapshot” of one night of sleep, whereas the PROs surveyed a 4-week time frame prior to that snapshot. Thus, these two measures provide discrete and fundamentally different assessments of sleep as has been proposed by others [34]. Therefore, we predicted that these two datasets might yield different associations with PNI parameters, providing the rationale for incorporating both measurement modalities into the clinical study. In order to compare these measures directly, we examined longitudinal change from time points 1 to 2.

By both measures, the population had a relatively normal range of sleep duration, averaging 6.6 h by actigraph scored sleep time and 6.8 h by PRO sleep quantity at T1, and 6.8 h and 6.8 h by the same measures, respectively, at T2, Supplemental Table 3. Correlations of longitudinal change from T1 to T2 in actigraph and PRO-assessed sleep parameters are presented as a matrix in Table 2. Interestingly, longitudinal changes in one sleep parameter from each method, actigraph scored sleep time and the self-reported SLP9, were inversely correlated (*r* = − 0.25, *p* < 0.05). Next, the relationships between sleep parameters were compared with measures of emotional distress.

**Table 2:** Correlation matrix of change (T2-T1) in actigraph and sleep PRO parameters

		Actigraph parameters (T2-T1)							Emotional distress (T2-T1)	
		Activity count	Efficiency	Wake time	% wake	Sleep time	% sleep	Sleep bouts	ED-depression	ED-anxiety
Sleep PRO parameters	Sleep disturbance	0.08	− 0.22^*^	0.14	0.16	− 0.13	− 0.16	− 0.10	0.23^**^	0.24^**^
	Snoring	− 0.09	− 0.09	− 0.11	− 0.15	0.04	0.15	0.04	0.10	0.04
	Shortness breath	− 0.05	− 0.06	− 0.05	0.01	− 0.20^*^	− 0.01	− 0.11	0.10	0.01
	Sleep adequacy	− 0.05	0.09	− 0.09	− 0.15	0.14	0.15	− 0.01	− 0.17	− 0.13^*^
	Daytime somnolence	− 0.04	0.07	− 0.04	− 0.09	− 0.01	0.09	0.02	− 0.03	0.06
	Sleep problems index	0.09	− 0.20	0.11	0.16	− 0.25^*^	− 0.16	− 0.10	0.29^**^	0.25^**^
	Sleep quantity	− 0.18	0.37^*^	− 0.13	− 0.18	0.24^*^	0.18	0.06	− 0.27^**^	− 0.21
Emotional Distress (T2-T1)	ED-depression	− 0.09	− 0.12	− 0.12	− 0.03	-0.32^**^	0.03	− 0.06		
	ED-anxiety	− 0.04	− 0.19	− 0.09	0.01	− 0.25^*^	− 0.01	− 0.20		

Spearman’s correlation coefficients of change over time (T2-T1) in sleep measures, *n* = 71

*AC* activity count

### The Relationship of Monitored and Self-Reported Sleep Measures in an Emotionally Distressed Population

The emotional distress subscales of depression and anxiety were included in the correlation matrix of Table 1 to show the relationship between changes in sleep parameters and changes in distress. Changes in depression and anxiety were associated with changes in the following sleep PRO: sleep disturbance (*r* = 0.23, *p* < 0.01 and *r* = 0.24, *p* < 0.01, respectively), the Sleep Problems Index (SLP9) (*r* = 0.29, *p* < 0.01 and *r* = 0.25, *p* < 0.01), and sleep quantity (*r* = − 0.27 and − 0.21). Amongst the actigraph parameters, only the change in sleep time correlated with change in both depression and anxiety (*r* = − 0.32, *p* < 0.01 and *r* = − 0.25, *p* < 0.05, respectively). Based on their relationship with emotional distress, the scored sleep time parameter from actigraph and the SLP9 from the PRO were chosen, as an objective and subjective measure, respectively, for comparison with cytokines. Sleep time and sleep duration are actigraph parameters that have been shown to correlate with self-reported sleep in other studies.

Participants were further divided into three subgroups based on change in sleep parameters. Groups included those with a decrease greater than or equal to one standard deviation (SD) from the mean, a change of less than one SD from the mean in either direction, or an increase greater than or equal to one SD from the mean. For those whose sleep time increased, both depression and anxiety decreased significantly (test for linear trend, *p* = 0.02 and *p* = 0.05 respectively). Conversely, for those who reported increased sleep problems on the SLP9, depression and anxiety increased (test for linear trend, *p* = 0.001 and *p* = 0.01, respectively).

### Changes in Actigraph Monitored Sleep Time are Associated with Changes in Inflammatory Cytokine Levels

**Fig. 1:**
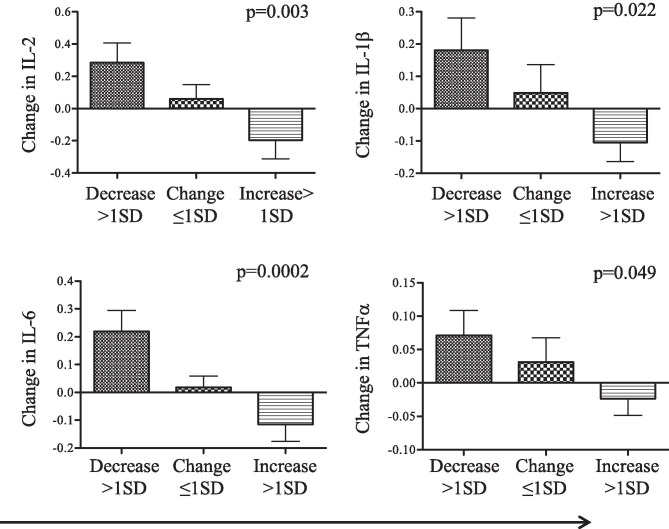
Acute-phase response cytokines decreased as sleep time increased. Categorical change (T2-T1) in actigraph scored sleep time was divided into three subgroups; a decrease greater than or equal to one standard deviation (SD) from the mean (*N* = 19), change less than one SD from the mean (*N* = 31), or an increase greater than or equal to one SD from the mean (*N* = 21). Linear trend tests performed against change in cytokines with correlations that reached or approached significance. The acute-phase cytokines IL-2 (*p* = 0.003), IL-1β (*p* = 0.022), IL-6 (*p* = 0.0002), and TNFα (*p* = 0.049) all significantly decreased as sleep time increased. *N* = 71, significance is set at *p* < 0.05

As noted above, there are well-established relationships between sleep and immune parameters. Since the nature of the clinical study involved the evaluation of circulating blood cytokine concentrations, as a measure of the state of the immune system, we examined longitudinal changes (T2-T1) in circulating cytokine levels in relation to longitudinal changes in sleep measures were examined. A multiplex assay covering several types of cytokines was chosen for this study, covering Th1, Th2, and pro-inflammatory cytokines. Changes in sleep time were significantly inversely correlated with changes in the pro-inflammatory cytokine, IL-6, and T cell proliferation factor, IL-2 (*r* = − 0.297, *p* = 0.002 and − 0.278, *p* = 0.005 respectively). Two other pro-inflammatory cytokines, IL-1β and TNFα, approached, but did not attain significance with a similar inverse correlation (*r* = − 0.22, *p* = 0.23 and − 0.20, *p* = 0.03, respectively). When subjects were classified by tertile of increasing longitudinal change in sleep time, all four pro-inflammatory cytokines demonstrated significant trends, with cytokines decreasing as sleep time increased (IL-2, *p* = 0.003; IL-1β, *p* = 0.022; IL-6, *p* = 0.0002; TNFα, *p* = 0.049) (Fig. 1). Other cytokines included in the panel (IL-4, IL-5, IL-10, IL-12p70, IL-13, and interferon gamma) showed no significant trends correlating with sleep time. As pro-inflammatory cytokines, particularly IL-1β and TNFα, are well characterized for their roles in sleep regulation, so it is expected that an increase in sleep time is associated with a systemic decrease in these proteins.

### Changes in Self-Reported Sleep Problems Over Time Correlate with Changes in other Cytokine Levels

**Fig. 2:**
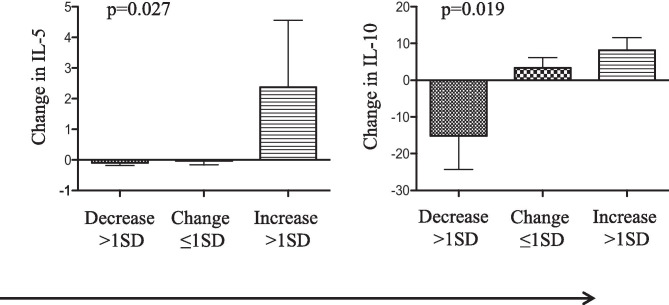
Th2 and immunosuppressive cytokines increased as sleep problems increased. Categorical change (T2-T1) in sleep problems (SLP9) was divided into three subgroups; a decrease greater than or equal to one standard deviation (SD) from the mean (*N* = 21), change less than one SD from the mean (*N* = 30), or an increase greater than or equal to one SD from the mean (*N* = 20). Linear trend tests performed against change in cytokines with correlations that reached or approached significance. Significant trends were found with IL-5 (*p* = 0.027) and IL-10 (*p* = 0.019). *N* = 71, significance is set at *p* < 0.05

Potential associations of longitudinal change in circulating cytokine levels and the SLP9 were also evaluated. Change in the SLP9 was significantly correlated with changes in the inflammatory cytokine TNFα (*r* = 0.28, *p* = 0.003), the Th2 cytokine IL-5 (*r* = 0.21, *p* = 0.03), which is most representative of highly polarized Th2 responses [16], and the counter-regulatory cytokine IL-10 (*r* = 0.22, *p* = 0.02). Interestingly, there were no significant correlations with changes in Th1 cytokines, IFNγ and IL-12, or the other Th2 cytokines, IL-4 and IL-13. Linear trend tests for change in these cytokines across increasing tertiles of longitudinal change in the SLP9 confirmed the significant positive correlations for changes in IL-5 and IL-10 (*p* = 0.027 and 0.016, respectively), but not for TNFα (*p* = 0.097) (Fig. 2).

## Discussion

Cervical cancer patients and survivors report higher than average levels of perceived stress, depression, and anxiety [35]. The reported association of sleep dysregulation with both distress and inflammation led us to hypothesize that these parameters are interrelated and that an improvement in one would be reflected in the other two [36–39]. This hypothesis was confirmed by the network of correlating changes in emotional distress, sleep quantity and quality, and circulating cytokines. Changes over time in these factors correlated in the expected manner, that is, an increase in scored sleep time or decrease in sleep problems correlated with a decrease in emotional distress and a decrease in pro-inflammatory or Th2 and counter-regulatory cytokines. Longitudinal changes in measures of sleep, both single night objective measures (actigraph scored sleep time) and aggregate subjective data (self-reported sleep problems), were found to be associated with changes in distress and circulating cytokines. Interestingly, change in monitored sleep time significantly correlated with changes in acute phase cytokines (IL-6, IL-2, IL-1β, and TNFα), while change in self-reported sleep problems is associated with changes in the Th2 cytokine IL-5 and the counter-regulatory cytokine IL-10.

The relationship between sleep duration, sleep quality, mood, and immune system is complex at best. This is complicated by the myriad diurnal patterns and two-way cross talk between all of these elements [40–44]. Recent reviews provide a detailed overview of what is currently known about the psychoneuroimmune axis as it relates to sleep and sleep disruptions [40–44]. It is noteworthy that these reviews acknowledge a lack of consistency in the experimental findings, and thus, some ambiguity surrounding what should or should not be expected. Our findings that change in sleep time was inversely associated with anxiety, depression, and serum cytokine levels (IL-2, IL-1β, IL-6, TNFα) are in keeping with the consensus of these reviews. Furthermore, our finding that increases in self-reported sleep problems were associated with higher measures of depression, anxiety, IL-5, and IL-10 are similarly consistent with the consensus of these reviews.

Further examination of these cytokines elucidates the role that they play in sleep regulation via the PNI axis. Pro-inflammatory response cytokines like TNFα, IL-1β, IL-2, and IL-6 decrease sleep time due to their role in the sleep homeostat [45]. TNFα and IL-1β stimulate NF-κB, which in turn promotes the production of TNFα and IL-1β in a positive feedback loop, as well as IL-2, IL-6, and other anti-somnogenic substances [46]. Sleep deprivation increases serum IL-6 and TNFα levels [47, 48], consistent with our findings of an inverse association with sleep duration. IL-10 and IL-1 are both increased with either sleep deprivation or sleep disturbances [40, 41], again consistent with our findings.

In this study, we also report a trend of decrease in the Th2 cytokine, IL-5, and Th2-like cytokine, IL-10, with a decrease in sleep problems. Although other Th2 cytokines, IL-4 and IL-13, have been associated with sleep impairment, there is no extensive literature on sleep in relation to these cytokines. Elevated IL-5 has been shown to correspond to fatigue and impaired sleep in allergic subjects [49]. The increase in Th2 profile with sleep deprivation or disruption is consistent with our findings of increased IL-5 seen in our studies associated with increased sleep problems. IL-10 has been shown to inhibit slow wave sleep in rodents [50], but reduced IL-10 was associated with poor sleep quality in hemodialysis patients [51].

Th2 cytokines have a protective effect in the acute phase of an inflammatory response driven by cortisol expression. Therefore, it may not be unusual to see these levels decrease in concert with a decrease in sleep problems due to emotional distress, which can trigger cortisol production [52]. Given the inherent differences in the designs for human clinical studies of sleep and associated immune parameters, we are reassured that our findings are not at odds with the current understanding of this system. The association of sleep disturbances with increased levels of depression and anxiety is extremely well documented [53–56] and our findings that decreased sleep duration and increased sleep problems are both associated with higher PRO levels of depression and anxiety are consistent with this state of the art.

There is a possibility that the difference in the type of cytokine related to each of the different sleep measures was due to the nature of the measures themselves. In this study, actigraphy captured a snapshot of one night of sleep, wherein disturbances would be reflected in the immediate release of cytokines into the circulation. In contrast, the PRO measurement reflects aggregate effects of sleep disturbances spanning 4 weeks and may therefore relate to downstream effects of persistent sleep disturbances, manifesting as a shift toward a Th2 immune environment. These contrasting measurement strategies, i.e., actigraph snapshot or a 4-week self-reported interval, could be considered analogous to acute vs. chronic psychological stress responses. Interestingly, we did see an inverse correlation between the changes in sleep time and sleep problems, in which sleep time increased as sleep problems decreased. A few studies have examined the relationship of both objective and subjective sleep measures to depression and quality of life and reported similar results [57–59]. We conclude that these two sleep measures evaluate inherently different variables and may be associated with different downstream biological responses. This makes the selection of sleep assessment modality critical for a given biobehavioral hypothesis.

Although these data were obtained in the context of a randomized biobehavioral clinical study, there are several limitations. The number of individuals with a full set of PRO data as well as usable biospecimens and actigraphs was limited, relative to the total study cohort, raising the possibility of imparting unintended bias. However, there were no significant differences between the groups of excluded and included groups of participants in any of the measured parameters by Mann Whitney testing, with the exception of actigraphy, the absence of which was the basis of exclusion (data not shown). The logistics of the study could only accommodate collection of actigraph data for a single night. Data collected over 7–14 consecutive nights has been shown to provide a fuller assessment of an individual’s sleep pattern, which could impart greater confidence in detected longitudinal changes [25, 60–62]. Actigraphy data is affected by the sensitivity and sampling capabilities of the device and the scoring algorithm used to analyze the data, all of which impact the sleep-wake scoring. We attempted to abrogate some of this variability by examining change over time, rather than direct comparison. Despite showing results for parameters such as percent wake and sleep and efficiency, those parameters have been shown to be problematic by actigraphy measurement. Although use of a more comprehensive sleep PRO could also have been advantageous, this was balanced against the burden of multiple PROs, addressing various behavioral elements and domains, which were integrated into the clinical study. Limited data may also mask some relationships. While we found changes in sleep time and problems related to changes in emotional distress, there may be relationships between sleep parameters and PROs that were not significant due to the restricted number of subjects or the limited nature of the study. Nevertheless, our data are consistent with the published body of work in multiple other disease states, suggesting that these limitations are unlikely to impact or invalidate the conclusions drawn herein.

This study is the first to document associations between longitudinal changes in (1) sleep parameters, (2) emotional distress, and (3) immune parameters specifically in cervical cancer survivors. Cancer patients and survivors present an especially at-risk population for PNI dysregulation [16, 63–67]. The physiological trauma of the disease and treatment, along with the psychological stress of a cancer diagnosis and fear of progression or recurrence, has a significant impact on overall well-being. Sleep disturbance is prevalent in cancer patients and survivors, and understanding of its connection with the PNI could produce more effective interventions and improve QOL. Although a few groups have published extensive studies on stress and sleep in the context of cancer [2, 68–71, 72–74], few studies have examined the relationship of sleep, distress, and immunity in cancer patients [75–77] or cancer survivors [2, 78, 79], and none have been focused on cervical cancer patients or survivors.

Cancer survivors are at risk for chronic distress and its associated physiological manifestations, such as sleep dysregulation and inflammatory immune responses. This study showed that in cervical cancer survivors, a longitudinal increase in sleep time or decrease in sleep problems corresponded with a reduction in self-reported emotional distress and attenuation of pro-inflammatory, Th2, and counter-regulatory cytokines. These data support the inclusion of sleep disruption in the biobehavioral paradigm and need for assessment thereof in clinical studies examining vulnerable cancer survivor populations.

## Supplementary Information

Below is the link to the electronic supplementary material.

Supplementary file1 (DOCX 39 KB)
