# Supplementary material for: Longitudinal Changes in Sleep: Associations with Shifts in Circulating Cytokines and Emotional Distress in a Cancer Survivor Population
Source: Int J Behav Med. 2021 Feb 17;28(1):140–50. doi: 10.1007/s12529-020-09950-0 (PMC7925505; doi:10.1007/s12529-020-09950-0)
Supplement: Supplementary file 1 — Supplementary file1 (DOCX 39 KB) [file 12529_2020_9950_MOESM1_ESM.docx]

**Supplemental Data**

Supplemental Table 1. Comparison of all measures by treatment group in longitudinal change from T1-T2.

| **T2-T1** | UC | | PTC | | Kruskal-Wallis post test | |
| --- | --- | --- | --- | --- | --- | --- |
|  | Mean | SD | Mean | SD | Diff Rank Sum | P<0.05 |
| sleep duration | 35.81 | 124.10 | -7.15 | 137.40 | 168.8 | No |
| total AC | 213.50 | 12040.00 | 1549.00 | 16788.00 | -95.87 | No |
| avg AC/min | -1.31 | 23.80 | 2.71 | 30.08 | -33.44 | No |
| avgAC/epoch | -2.37 | 30.74 | -1.09 | 27.98 | -12.4 | No |
| Std AC | -3.30 | 78.64 | 0.16 | 82.17 | -16.92 | No |
| Efficiency | 2.65 | 13.15 | -0.83 | 14.02 | 41.25 | No |
| wake time | 0.56 | 43.58 | -3.50 | 52.30 | 36.9 | No |
| % wake | -0.68 | 9.05 | -0.48 | 9.52 | -12.9 | No |
| sleep time | 33.72 | 126.00 | -5.68 | 131.40 | 114.4 | No |
| % sleep | 0.68 | 9.05 | 0.48 | 9.52 | 12.39 | No |
| # sleep bouts | 1.91 | 19.21 | 0.75 | 22.90 | 4.353 | No |
| avg sleep bouts | 13.42 | 70.68 | 7.25 | 102.60 | 39.94 | No |
| IFNg | -0.15 | 8.18 | -1.45 | 16.42 | 3.84 | No |
| IL-12 | 0.29 | 10.17 | -1.53 | 9.71 | 259.00 | No |
| IL-2 | 0.19 | 2.36 | -3.02 | 26.20 | 141.00 | No |
| IL-1B | -0.04 | 0.67 | -0.32 | 2.82 | 39.45 | No |
| IL-6 | 0.64 | 4.67 | 1.36 | 11.63 | 77.74 | No |
| TNFa | 1.27 | 5.03 | 0.47 | 4.17 | 67.11 | No |
| IL-4 | -0.10 | 16.15 | -3.08 | 23.01 | 222.30 | No |
| IL-5 | 0.10 | 0.78 | 0.10 | 2.35 | 108.10 | No |
| IL-13 | 0.61 | 3.15 | -0.41 | 6.36 | 109.20 | No |
| IL-10 | 0.80 | 21.16 | -1.48 | 16.40 | 169.60 | No |
| FACTG | 2.73 | 11.08 | 2.11 | 11.57 | -55.41 | No |
| FACTGS | 1.32 | 5.61 | 0.62 | 8.92 | -42.39 | No |
| FACTCx | 3.85 | 13.64 | 4.84 | 15.42 | -146.50 | No |
| ED-DepTS | -0.28 | 9.40 | -3.44 | 8.38 | 377.70 | No |
| ED-AnxTS | -1.37 | 9.68 | -2.92 | 9.50 | 230.40 | No |
| GPC_tot | -0.15 | 5.66 | -2.19 | 6.14 | 345.30 | No |
| BSI-GSI-S | -1.63 | 8.58 | -3.49 | 9.65 | 187.70 | No |
| SLPD4 | -1.83 | 18.44 | -2.88 | 15.76 | 19.02 | No |
| SLPSNR1 | 0.00 | 25.90 | -7.27 | 25.41 | 59.00 | No |
| SLPSOB1 | -5.88 | 25.36 | 5.95 | 22.54 | -85.95 | No |
| SLPA2 | 2.57 | 20.05 | 0.54 | 26.97 | 12.25 | No |
| SLPS3 | -2.48 | 17.49 | -3.07 | 16.38 | 24.31 | No |
| SLP6 | -3.31 | 15.68 | -1.26 | 12.79 | -22.57 | No |
| SLP9 | -2.08 | 13.85 | -1.10 | 12.51 | -18.89 | No |
| SLPQRAW | 0.17 | 1.32 | -0.04 | 1.03 | 12.24 | No |

Results are differences in rank sum by Dunn’s Multiple Comparison Test of Kruskal-Wallis. At T1, n=71.Abbreviations: avg, average; AC, standardized activity count; min, minute; Std, standardized; : IFNg, interferon gamma; TNFa, tumor necrosis factor alpha; FACTG, Functional Assessment of Cancer Therapy – General; FACTGS, FACTG standardized; FACTCx, FACT Cervix; ED-D, emotional distress-depression; ED-A, emotional distress-anxiety; GPC, global perceived change; BSI-GSI-S, brief symptom inventory-global severity index-standardized; SLPD4, sleep disturbance; SNR1, Snoring; SOB1, shortness of breath; SLPA2, sleep adequacy; SLPS3, daytime somnolence; SLP9, sleep problems index; SLPQ, sleep quantity.

Supplemental Table 2. Comparison of all measures by treatment group at T1.

| **T1** | UC | | PTC | | Kruskal-Wallis post test | |
| --- | --- | --- | --- | --- | --- | --- |
|  | Mean | SD | Mean | SD | Diff Rank Sum | P<0.05 |
| sleep duration | 423.20 | 98.40 | 462.00 | 93.47 | -19.25 | No |
| total AC | 7863.00 | 6712.00 | 8853.00 | 7686.00 | -1.08 | No |
| avg AC/min | 18.05 | 14.14 | 18.65 | 14.52 | -8.47 | No |
| avgAC/epoch | 16.52 | 17.80 | 13.74 | 24.42 | 85.80 | No |
| Std AC | 63.72 | 55.62 | 54.94 | 61.86 | 97.37 | No |
| Efficiency | 78.24 | 11.95 | 79.82 | 8.63 | -18.39 | No |
| wake time | 50.40 | 31.16 | 51.95 | 36.08 | 5.53 | No |
| % wake | 11.66 | 6.44 | 11.03 | 6.65 | 17.94 | No |
| sleep time | 375.00 | 87.51 | 410.00 | 86.61 | -14.86 | No |
| % sleep | 88.34 | 6.44 | 88.97 | 6.65 | -3.51 | No |
| # sleep bouts | 27.03 | 12.17 | 32.34 | 12.31 | -69.06 | No |
| avg sleep bouts | 16.16 | 6.70 | 17.47 | 19.52 | 15.21 | No |
| IFNg | 10.21 | 13.07 | 15.98 | 29.75 | -53.50 | No |
| IL-12 | 6.07 | 9.02 | 13.75 | 24.19 | -130.90 | No |
| IL-2 | 2.90 | 4.79 | 18.54 | 69.42 | -134.10 | No |
| IL-1B | 1.15 | 2.28 | 3.23 | 12.07 | -47.39 | No |
| IL-6 | 4.75 | 6.96 | 8.40 | 13.24 | -94.97 | No |
| TNFa | 9.71 | 6.72 | 12.66 | 13.01 | -33.42 | No |
| IL-4 | 22.49 | 38.10 | 38.76 | 65.80 | -162.30 | No |
| IL-5 | 0.95 | 1.29 | 1.94 | 3.83 | -53.86 | No |
| IL-13 | 2.39 | 4.08 | 7.96 | 21.78 | -135.00 | No |
| IL-10 | 17.61 | 25.13 | 31.92 | 56.26 | -105.20 | No |
| FACTG | 78.97 | 17.96 | 85.31 | 15.04 | -59.60 | No |
| FACTGS | 58.59 | 8.51 | 61.87 | 10.68 | -38.03 | No |
| FACTCx | 122.80 | 23.03 | 129.20 | 21.67 | -8.63 | No |
| ED-DepTS | 53.84 | 9.30 | 51.03 | 8.93 | 36.98 | No |
| ED-AnxTS | 53.73 | 11.10 | 49.84 | 10.05 | 50.94 | No |
| GPC_tot | 19.45 | 6.95 | 21.77 | 8.44 | -35.23 | No |
| BSI-GSI-S | 51.27 | 9.82 | 48.95 | 11.92 | 29.48 | No |
| SLPD4 | 40.43 | 29.96 | 33.73 | 27.1 | 153.40 | No |
| SLPSNR1 | 37.42 | 31.3 | 28.57 | 31.91 | 255.30 | No |
| SLPSOB1 | 28.13 | 29.99 | 9.474 | 17.85 | 616.40 | No |
| SLPA2 | 50 | 24.11 | 51.32 | 27.82 | 12.27 | No |
| SLPS3 | 31.71 | 22.11 | 24.39 | 19.92 | 163.30 | No |
| SLP6 | 38.14 | 17.68 | 32.54 | 21.15 | 158.70 | No |
| SLP9 | 38.98 | 20.06 | 32.12 | 21.48 | 167.90 | No |
| SLPQRAW | 6.614 | 1.158 | 6.684 | 1.477 | 0.01 | No |

Results are differences in rank sum by Dunn’s Multiple Comparison Test of Kruskal-Wallis. At T1, n=71.Abbreviations: avg, average; AC, standardized activity count; min, minute; Std, standardized; : IFNg, interferon gamma; TNFa, tumor necrosis factor alpha; FACTG, Functional Assessment of Cancer Therapy – General; FACTGS, FACTG standardized; FACTCx, FACT Cervix; ED-D, emotional distress-depression; ED-A, emotional distress-anxiety; GPC, global perceived change; BSI-GSI-S, brief symptom inventory-global severity index-standardized; SLPD4, sleep disturbance; SNR1, Snoring; SOB1, shortness of breath; SLPA2, sleep adequacy; SLPS3, daytime somnolence; SLP9, sleep problems index; SLPQ, sleep quantity.

Supplemental Table 3. Comparison of all measures by treatment group at T2.

| **T2** | UC | | PTC | | Kruskal-Wallis post test | |
| --- | --- | --- | --- | --- | --- | --- |
|  | Mean | SD | Mean |  | Mean | SD |
| sleep duration | 439.7 | 86.11 | 474.4 | 108 | -12.02 | No |
| total AC | 8014 | 5814 | 8748 | 5769 | -18.52 | No |
| avg AC/min | 17.43 | 11.57 | 19.45 | 14.83 | -45.3 | No |
| avgAC/epoch | 8.748 | 5.663 | 15.39 | 20.73 | -99.12 | No |
| Std AC | 42.44 | 22.15 | 59.17 | 45.74 | -112.1 | No |
| Efficiency | 79.97 | 9.012 | 79.18 | 12.17 | 11.06 | No |
| wake time | 47.25 | 29.13 | 54.4 | 40.32 | -45.76 | No |
| % wake | 10.34 | 5.421 | 11.77 | 8.428 | -23.99 | No |
| sleep time | 392.4 | 72.69 | 419.9 | 107.1 | -11.11 | No |
| % sleep | 89.66 | 5.421 | 88.23 | 8.428 | 10.42 | No |
| # sleep bouts | 34.13 | 18.43 | 29.33 | 13.79 | 48.5 | No |
| avg sleep bouts | 28.51 | 79.52 | 31.25 | 92.32 | -50.04 | No |
| IFNg | 10.38 | 10.8 | 13.9 | 17.81 | -48.13 | No |
| IL-12 | 7.374 | 12.58 | 14.07 | 24.8 | -122.4 | No |
| IL-2 | 2.875 | 3.455 | 12.14 | 36.48 | -275.3 | No |
| IL-1B | 0.9823 | 1.651 | 2.499 | 8.151 | -40.34 | No |
| IL-6 | 5.529 | 6.445 | 10.28 | 18.39 | -88.41 | No |
| TNFa | 10.07 | 7.454 | 13.79 | 13.98 | -51.31 | No |
| IL-4 | 25.12 | 34.88 | 36.63 | 60.15 | -84.27 | No |
| IL-5 | 1.05 | 1.477 | 2.306 | 5.375 | -41.69 | No |
| IL-13 | 2.847 | 4.154 | 6.461 | 18.29 | -83.47 | No |
| IL-10 | 20.24 | 24.11 | 32.06 | 49 | -81.09 | No |
| FACTG | 81.9 | 14.51 | 87.6 | 16.69 | -36.26 | No |
| FACTGS | 59.3 | 7.748 | 63.5 | 11.33 | -41.43 | No |
| FACTCX | 126.8 | 20.7 | 134 | 24.12 | -2.773 | No |
| ED-DepTS | 50.58 | 8.952 | 49.24 | 10.85 | 20.64 | No |
| ED-AnxTS | 51.85 | 10.96 | 48.11 | 9.218 | 46.44 | No |
| GPC_tot | 19.42 | 8.078 | 18.78 | 8.162 | 11.11 | No |
| BSI-GSI-S | 50.76 | 12.75 | 46.39 | 13.03 | 51.73 | No |
| SLPD4 | 38.54 | 26.47 | 30.18 | 26.46 | 212.90 | No |
| SLPSNR1 | 38.18 | 32.16 | 20.54 | 27.68 | 492.50 | No |
| SLPSOB1 | 23.03 | 32.06 | 15.26 | 25.65 | 205.40 | No |
| SLPA2 | 53.33 | 24.07 | 52.37 | 30.08 | 99.78 | No |
| SLPS3 | 29.08 | 23.08 | 21.05 | 21.28 | 182.00 | No |
| SLP6 | 34.43 | 20.33 | 30.96 | 22.36 | 118.90 | No |
| SLP9 | 36.63 | 20.98 | 30.54 | 22.69 | 158.70 | No |
| SLPQRAW | 6.788 | 1.159 | 6.625 | 1.604 | 12.49 | No |

Results are differences in rank sum by Dunn’s Multiple Comparison Test of Kruskal-Wallis at T2, n=71. Abbreviations: avg, average; AC, standardized activity count; min, minute; Std, standardized; : IFNg, interferon gamma; TNFa, tumor necrosis factor alpha; FACTG, Functional Assessment of Cancer Therapy – General; FACTGS, FACTG standardized; FACTCx, FACT Cervix; ED-D, emotional distress-depression; ED-A, emotional distress-anxiety; GPC, global perceived change; BSI-GSI-S, brief symptom inventory-global severity index-standardized; SLPD4, sleep disturbance; SNR1, Snoring; SOB1, shortness of breath; SLPA2, sleep adequacy; SLPS3, daytime somnolence; SLP9, sleep problems index; SLPQ, sleep quantity.
